# Diffusion-Weighted MRI as a Non-Invasive Diagnostic Tool for Ascites Characterization: A Comparative Analysis of Mean and Minimum ADC Values Against the Serum-Ascites Albumin Gradient

**DOI:** 10.3390/diagnostics15243130

**Published:** 2025-12-09

**Authors:** Abdullah Enes Ataş, Şeyma Ünüvar, Hasan Eryeşil, Naile Kökbudak

**Affiliations:** 1 Department of Radiology, Necmettin Erbakan University, Konya 42090, Türkiye; 2 Department of Radiology, Konya City Hospital, Konya 42020, Türkiye; seymababaoglu@hotmail.com; 3 Department of Radiology, Tatvan State Hospital, Bitlis 13200, Türkiye; hsneryesil@gmail.com; 4 Department of Pathology, Necmettin Erbakan University, Konya 42090, Türkiye; nkokbudak@erbakan.edu.tr

**Keywords:** ascites, peritoneal neoplasms, magnetic resonance imaging, diffusion weighted imaging, paracentesis

## Abstract

**Background/Objectives**: This study aimed to evaluate the diagnostic accuracy of Apparent Diffusion Coefficient (ADC) values, derived from Diffusion-Weighted Imaging (DWI), in differentiating benign and malignant ascites. **Methods**: This retrospective study included 150 patients (85 benign, 65 malignant) who underwent abdominal MRI. All patients were scanned on a DWI sequence (b-values: 0, 500, and 1000 s/mm^2^). Two experienced radiologists, blinded to clinical and cytological outcomes, measured the mean ADC (ADCmean) from three distinct ROIs and the minimum ADC (ADCmin) from the area of lowest signal intensity on the ADC map. The diagnostic performance of ADC parameters and the Serum-Ascites Albumin Gradient (SAAG) was assessed using Receiver Operating Characteristic (ROC) curve analysis. **Results**: The mean values of ADCmean (3162 ± 204 × 10^−6^ mm^2^/s) and ADCmin (2885 ± 148 × 10^−6^ mm^2^/s) in the malignant group were significantly lower than those in the benign group (3596 ± 239 and 3322 ± 218 × 10^−6^ mm^2^/s; *p* = 0.006 and *p* = 0.0016, respectively). Inter-observer agreement was good for both ADCmean (ICC = 0.844) and ADCmin (ICC = 0.879). In the ROC analysis, ADCmin demonstrated the highest diagnostic performance (AUC: 0.930). An optimal cut-off value for ADCmin of ≤ 2983 × 10^−6^ mm^2^/s yielded 81.5% sensitivity and 85.8% specificity. The diagnostic performance of ADCmin was found to be superior to that of ADCmean (AUC: 0.877) and SAAG (AUC: 0.919). **Conclusions**: ADC values derived from DWI, particularly ADCmin, represent a highly accurate, non-invasive, and reproducible biomarker for differentiating benign from malignant ascites. The identified ADCmin threshold provides quantitative parameter that can aid in patient triage, especially when cytology is inconclusive, potential surrogate for fluid characterization.

## 1. Introduction

Ascites, defined as the abnormal accumulation of fluid within the peritoneal cavity resulting from various benign and malignant pathologies, serves as a significant prognostic indicator of the underlying disease [1]. The accurate and rapid determination of ascites etiology is of critical importance for establishing patient management and treatment strategies. Malignant ascites accounts for approximately 10% of cases and typically manifests as a symptom of advanced-stage malignancies, such as ovarian, breast, colon, gastric, and pancreatic cancers, and is associated with a poor prognosis [2].

Currently, the gold standard method for the differential diagnosis of ascites is diagnostic paracentesis, an invasive procedure, followed by the cytological and biochemical analysis of the obtained fluid [2,3]. However, this method has significant limitations. The sensitivity of cytological examination can be suboptimal, remaining as low as 40–60%, particularly in certain tumor types and in patients with a low tumor burden [1]. This situation can lead to diagnostic delays, repeated invasive procedures, and false-negative results, consequently hindering the patient’s access to appropriate treatment. Although biochemical markers, such as the serum-ascites albumin gradient (SAAG), are useful in distinguishing transudative ascites due to portal hypertension from exudative causes, their ability to differentiate among various exudative etiologies, including malignancy, is limited [4]. Furthermore, these methods do not provide direct information regarding the physical microstructure or cellular composition of the fluid.

In recent years, imaging techniques that provide functional information regarding tissue microstructure have gained prominence in oncology. One of these techniques, Diffusion-Weighted Magnetic Resonance Imaging (DWI), is a functional imaging modality that non-invasively measures the microscopic thermal (Brownian) motion of water molecules within tissues [5,6]. The Apparent Diffusion Coefficient (ADC), a quantitative parameter of this technique, numerically expresses the mobility of water molecules and offers valuable insights into the tissue microenvironment [7].

The underlying principle of ADC is based on the restrictive effect of tissue microstructure on diffusion. Tissues characterized by high cellularity, narrowed extracellular spaces, and disrupted cell membranes impede the free motion of water molecules. This phenomenon manifests as high signal intensity on DWI and low quantitative values on ADC maps [8]. This principle has been successfully employed for the differentiation of malignant and benign lesions in numerous organs, including the liver [9], breast [10], endometrium, and soft tissue tumors [11]. In addition to malignancy differentiation, DWI has also been reported to be utilized in some inflammatory diseases to distinguish active disease from chronic conditions [12].

This concept has recently been extended to the characterization of body fluids [13,14,15]. A limited number of preliminary studies have suggested that malignant intraperitoneal fluids may exhibit lower ADC values compared to benign transudative fluids [16]. The underlying pathophysiological mechanism for this diffusion restriction is hypothesized to stem from the presence of tumor cells, cellular debris, elevated protein concentration, and consequently, increased viscosity within the malignant ascites [17]. However, the current literature predominantly consists of small patient cohorts and exhibits heterogeneity in terms of ADC measurement methodology [16,18].

In this context, ADC measurement extends beyond being merely a diagnostic parameter, possessing the potential to act as an “imaging-based liquid biopsy” that reflects the biophysical properties of the ascitic fluid. While conventional liquid biopsy provides information at the molecular level, ADC quantitatively assesses the physical microstructure of the fluid in its entirety, such as its overall cellularity and viscosity. This provides non-invasive, instantaneous, and global information about the fluid’s composition, potentially offering a window into the biological activity of the disease.

Moreover, DWI may offer a solution to the “sampling error” problem inherent in paracentesis. Whereas paracentesis involves acquiring a small fluid sample from a single point within the peritoneal cavity, DWI scans the entire peritoneal cavity. Thereby, the risk of false-negative cytology results arising from the heterogeneous distribution of tumor cells can be mitigated [19]. The ADC map, by identifying regions potentially harboring the highest tumor cell concentration (areas with the lowest ADC values), may even provide a roadmap for targeted paracentesis.

The hypothesis of this study is that malignant ascites, owing to its high cellular and proteinaceous components, will significantly restrict the diffusion of water molecules compared to benign ascites, and that this restriction can be quantitatively detected as a decrease in ADC values measured by DWI. The aim of this study is to retrospectively evaluate the diagnostic accuracy of mean and minimum ADC values obtained with DWI for the differentiation of benign and malignant ascites in a large patient cohort; to determine an optimal diagnostic threshold value; and to compare the performance of this non-invasive method with traditional biochemical markers.

## 2. Materials and Methods

### 2.1. Study Design and Patient Population

This retrospective study was conducted in accordance with the principles of the Declaration of Helsinki and received approval from the Necmettin Erbakan University Non-Pharmaceutical and Non-Medical Device Research Ethics Committee (Decision date: 26 September 2025; Approval No: 2025-5986). Informed consent was obtained from all subjects involved in the study.

Between 1 January 2020, and 1 January 2025, 185 patients were initially screened. 35 patients were excluded due to insufficient fluid volume (*n* = 20), or severe MRI artifacts (*n* = 15).

150 patients who presented to our clinic due to intra-abdominal free fluid (ascites) and were scheduled for abdominal Magnetic Resonance Imaging (MRI) were included in the study.

Adult patients with measurable ascites on MRI were enrolled, excluding those with MRI contraindications, complex fluid features (such as hemorrhagic ascites), recent intraperitoneal chemotherapy, or non-diagnostic image quality. The study cohort was subsequently stratified into two groups based on etiology: a benign group (n = 85) comprising conditions such as liver cirrhosis and heart failure confirmed via clinical and imaging evaluation, and a malignant group (n = 65) verified through cytological or histopathological analysis of malignancies. For patients in the benign group without histopathological confirmation, the diagnosis was established based on clinical stability or regression of ascites during a follow-up period of at least 6 months, in conjunction with supportive laboratory findings.

Demographic data, primary diagnosis, and significant comorbidities were recorded for all patients from the hospital information management system using a standardized form. Albumin, lactate dehydrogenase (LDH), and glucose levels were measured from blood samples and ascitic fluid samples (obtained via paracentesis) collected within one week prior to the MRI examination. The serum-ascites albumin gradient (SAAG) was calculated by subtracting the ascitic fluid albumin value from the serum albumin value (SAAG = Albumin _serum_ − Albumin _ascites_ g/dL) [20]. Sampling for serum and ascitic fluid albumin levels was performed on the same day.

### 2.2. MRI Protocol and Radiologic Evaluation

Imaging was performed using a 1.5 Tesla (1.5T) MRI system (Siemens Magnetom Aera, Erlangen, Germany) equipped with an 18-channel body coil. In addition to the standard abdominal MRI protocol (comprising axial T2-weighted HASTE and axial/coronal T1-weighted VIBE sequences), an axial, respiratory-triggered, spectrally fat-suppressed, single-shot echo-planar (SS-EPI) DWI sequence was acquired. Motion artifacts arising from respiration and peristalsis are the most significant factors affecting measurement accuracy in abdominal DWI. In this study, to minimize motion artifacts and thereby obtain high signal-to-noise ratio (SNR) and reliable ADC values, a respiratory-triggered sequence was employed, which acquires data during the most stable phase of the respiratory cycle (25). The DWI sequence parameters were as follows: Repetition Time (TR)/Echo Time (TE): 4500/65 ms; slice thickness: 6 mm; slice gap: 1 mm; Field of View (FOV): 380 × 380 mm; matrix: 128 × 128. Diffusion gradients were applied in three orthogonal directions using b-values of 0, 500, and 1000 s/mm^2^. The selection of these b-values was based on established applications in the literature, intending to both minimize perfusion-related effects (at low b-values) and maximize diffusion-weighting (at high b-values) to enhance tissue contrast. ADC maps were automatically generated by the scanner’s intrinsic software using a mono-exponential model.

**Figure 1:**
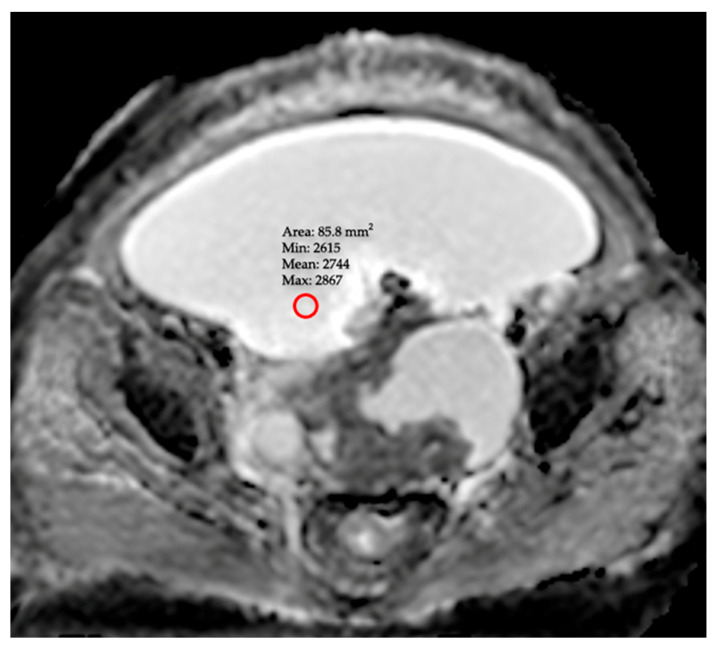
DWI MRI image in a patient with ovarian cancer, demonstrating a ROI placed within the intra-abdominal free fluid for ADC measurement (red circle).

All MRI images were independently evaluated on a Picture Archiving and Communication System (PACS) workstation by two radiologists (A.E.A. and Ş.Ü.) with 9 and 8 years of experience in abdominal radiology, respectively. The reviewers were blinded to each other’s findings and to the patients’ clinical and cytological outcomes. ADC measurements were performed directly on the ADC maps. For each patient, circular Regions of Interest (ROIs) were placed in at least three distinct anatomical locations where the ascitic fluid appeared most homogeneous and distant from potential artifact sources, such as blood vessels or bowel loops (e.g., the right subhepatic space, the left paracolic gutter, and the pelvic pouch of Douglas) (Figure 1). The ROI size was standardized, ranging from 50 to 100 mm^2^, to ensure consistency across measurements. This multi-ROI approach was intended to mitigate potential sampling bias and provide a more representative value reflective of the fluid’s global characteristics.

The quantitative parameters measured were as follows:

• ADCmean (Mean ADC): The arithmetic mean of the ADC values obtained from the three distinct ROIs.
• ADCmin (Minimum ADC): A value obtained from a single ROI placed on the area exhibiting the lowest signal intensity (appearing darkest) on the ADC map, following a meticulous inspection of the entire peritoneal cavity. To prevent this measurement from being subjective, observers used standard window settings (window width/level). This approach is predicated on the hypothesis that it may reflect the most aggressive component of the pathology by specifically targeting the region of highest focal malignancy or greatest fluid viscosity.
• Inter-observer agreement was also calculated to determine the reliability of these measurements.

### 2.3. Statistical Analysis

Minimum sample size calculation was performed using G*Power software (v3.1, Düsseldorf, Germany). Based on a moderate effect size (Cohen’s d = 0.50), an alpha level (α) of 0.05, and a statistical power (1-β) of 0.80, the analysis indicated that a total of 128 patients (64 in the benign group and 64 in the malignant group) would be required.

The conformity of continuous variables to a normal distribution was evaluated using histogram analysis and the Shapiro–Wilk test. For intergroup comparisons, the Independent Samples t-test was used for normally distributed data, and the Mann–Whitney U test was applied for non-normally distributed data. The Chi-square test was utilized for the analysis of categorical variables. Inter-observer agreement for the ADCmean and ADCmin measurements was assessed using the Intraclass Correlation Coefficient (ICC). ICC values were interpreted as follows: <0.50 as poor, 0.50–0.75 as moderate, 0.75–0.90 as good, and >0.90 as excellent agreement. Receiver Operating Characteristic (ROC) curve analysis was performed to evaluate the diagnostic performance of the ADC parameters and SAAG in the differentiation of benign and malignant ascites. The Area Under the Curve (AUC) was calculated with a 95% confidence interval (CI). The optimal cut-off value for each parameter, along with the corresponding sensitivity, specificity, positive predictive value (PPV), and negative predictive value (NPV), was determined using the Youden index (Sensitivity + Specificity − 1). *p* value < 0.05 was considered statistically significant. Statistical analyses were performed using IBM SPSS v24.0 (Armonk, NY, USA) and Jamovi v2.6 (Sydney, Australia) software.

## 3. Results

### 3.1. Demographic and Clinical Characteristics

Of the 150 patients included in the study, 85 (56.7%) were in the benign ascites group (Group 1), and 65 (43.3%) were in the malignant ascites group (Group 2). The mean age of the entire study population was 65.3 ± 6.6 years, and 46.6% (n = 70) were female. There was no statistically significant difference between the two groups in terms of age (mean 62.4 ± 11.2 vs. 64.8 ± 9.5 years, respectively; *p* = 0.094) or sex distribution (48.2% female vs. 56.9%, respectively; *p* = 0.378). This indicates that the two groups were comparable regarding baseline demographic characteristics. The demographic data and the distribution of primary etiologies for the groups are summarized in Table 1.

### 3.2. Laboratory Findings

Statistically significant differences were observed when serum and ascitic fluid biochemical parameters were compared between the benign and malignant ascites groups. In the malignant ascites group, ascitic fluid LDH and ascitic fluid albumin levels were significantly higher (*p* < 0.001), whereas the SAAG value was significantly lower (*p* < 0.001), compared to the benign group. These findings corroborate the exudative character of malignant ascites and the transudative nature of the majority of benign ascites. The comparative results are presented in Table 2.

### 3.3. Inter-Observer Agreement

The agreement between the ADC measurements, performed independently by the two radiologists, was analyzed to assess the reproducibility of the method. A good level of inter-observer agreement was determined for both ADCmean measurements (Intraclass Correlation Coefficient [ICC] = 0.844, 95% CI: 0.801–0.879) and ADCmin measurements (ICC = 0.879, 95% CI: 0.844–0.906). This high agreement indicates that the ADC measurement methodology is standardized and reliable, with minimal observer-dependent variability.

**Figure 2:**
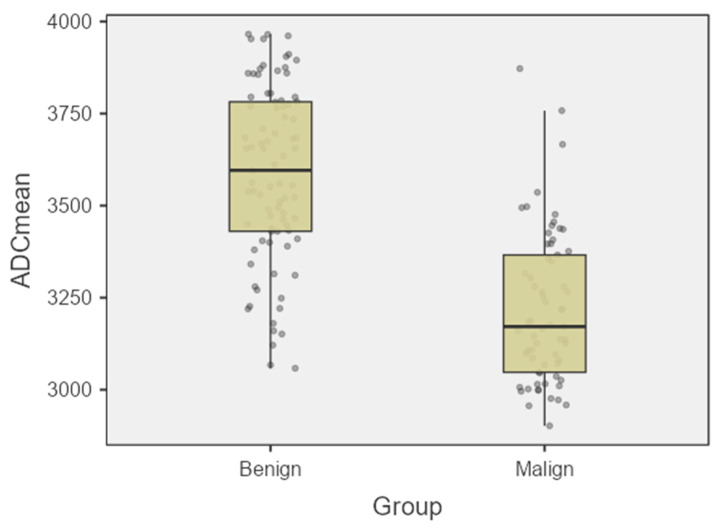

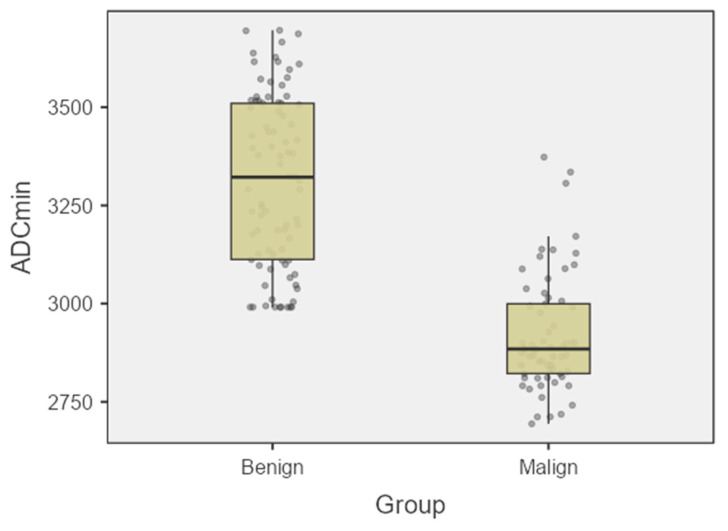
Box plot illustrating the distribution of ADCmean and ADCmin values in the benign and malignant ascites groups.

Measurement reproducibility was assessed using the coefficient of variation (CV). The calculated CVs were 2.0% for ADCmean and 1.7% for ADCmin, demonstrating high precision in the ADC measurements. Additionally, outliers were analyzed using the box-plot method (Figure 2), and it was observed that the three detected outliers did not significantly alter the overall results.

### 3.4. Diagnostic Performance Analysis of ADC Values

The findings testing the main hypothesis of the study revealed a highly distinct and statistically significant difference in ADC values between the malignant and benign ascites groups (Figure 2).

The mean of ADCmean value (3162 ± 204 × 10^−^^6^ mm^2^/s) and the mean ADCmin value (2885 ± 148 × 10^−6^ mm^2^/s) measured in the malignant ascites group were statistically significantly lower (*p* = 0.006 and *p* = 0.0016, respectively) than the values measured in the benign ascites group (3596 ± 239 × 10^−6^ mm^2^/s and 3322 ± 218 × 10^−6^ mm^2^/s, respectively). A detailed comparison of the ADC values between the groups is provided in Table 3.

**Figure 3:**
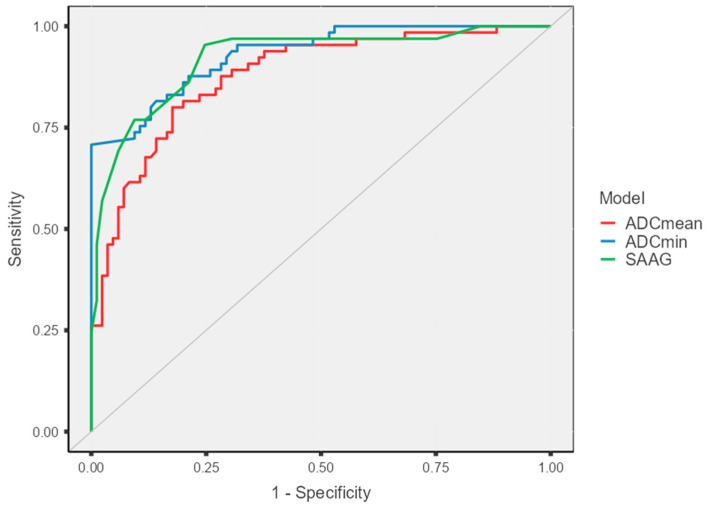
ROC curves demonstrating the diagnostic performance of ADCmean, ADCmin, and SAAG in the detection of malignant ascites.

ROC analysis, conducted to assess the diagnostic performance of ADCmin, ADCmean and SAAG in differentiating benign and malignant ascites, demonstrated the superior diagnostic accuracy of the ADC parameters, particularly ADCmin. The highest diagnostic performance was exhibited by ADCmin, achieving an Area Under the Curve (AUC) of 0.930 (95% CI: 0.892–0.968). The optimal cut-off value for ADCmin in detecting malignancy was determined to be 2983 × 10^−6^ mm^2^/s using the Youden index. At this threshold, the method provided 81.5% sensitivity, 85.8% specificity, an 81.5% positive predictive value (PPV), and an 85.8% negative predictive value (NPV). The Youden index was measured at 0.67. Although the AUC value for ADCmean (0.877, 95% CI: 0.822–0.932) and the AUC value for SAAG (0.919, 95% CI: 0.874–0.965) were also statistically significant, they were found to be lower than the diagnostic performance of ADCmin. The ROC curves are illustrated in Figure 3, and the diagnostic performance metrics are summarized in Table 4.

In the multivariate logistic regression analysis including ADCmin, SAAG, and age, ADCmin remained an independent predictor of malignancy (Odds Ratio: 0.81, 95% CI: 0.70–0.93, *p* = 0.004).

## 4. Discussion

The key finding of the present study is the highly statistically significant reduction in both ADCmean and ADCmin values in patients with malignant ascites compared to those with benign etiologies. This aligns with the established pathophysiological principle that malignant effusions, characterized by higher cellularity, increased protein content, and greater viscosity, inherently restrict the random Brownian motion of water molecules, thereby lowering the measured ADC values [21].

This study identifies an optimal ADCmin threshold (≤2983 × 10^−6^ mm^2^/s) that offers robust diagnostic accuracy. As a quantitative, non-invasive parameter, this threshold enhances radiologists’ diagnostic confidence, thereby aiding patient triage for either conservative management or more aggressive interventions, such as peritoneal biopsy.

Our findings corroborate and extend the results of previous studies investigating the utility of ADC measurement in ascitic fluid. For instance, Ștefan et al. [18] reported that malignant intraperitoneal fluids exhibit significantly lower ADC values compared to benign fluids. The mean ADC values reported in their study (approximately 3.543 × 10^−3^ mm^2^/s for benign and 3.057 × 10^−3^ mm^2^/s for malignant) were found to be comparable to those obtained in the present study. The concordance of these measurements, notwithstanding methodological differences such as the use of different MRI vendor systems, variations in MR technique (different b-values), or divergent ROI placement strategies reinforces the fundamental finding upon which our study is based. We believe a distinct contribution of our work is the utilization of the ADCmin parameter, which targets the most cellular and viscous component of the free fluid. This methodological approach enhanced the diagnostic power of ADC and allowed us to obtain values that correlate more closely with the underlying pathology [18].

SAAG reflects the oncotic pressure gradient. High SAAG (>1.1 g/dL) indicates portal hypertension, while low SAAG (<1.1 g/dL) typically indicates malignancy due to increased capillary permeability [20,22].

The significance of ADC measurement alongside traditional markers, such as the SAAG, in the differential diagnosis of benign and malignant ascites underscores the clinical potential of this method. In our study, both ADCmean and ADCmin values demonstrated diagnostic accuracy comparable to that of SAAG, one of the current non-invasive standards. This finding suggests that ADC measurement could serve as an alternative non-invasive diagnostic tool. Cytology, the gold standard, possesses significant limitations; although highly specific, it is invasive and suffers from low sensitivity, particularly in the presence of a low tumor burden [23,24]. In this context, DWI may play a critical role by offering a complementary, second-line evaluation when cytology is negative despite high clinical suspicion, or by providing a primary non-invasive modality for patients who face significant risks from paracentesis, including those with conditions like a bleeding diathesis [1,2].

In clinical practice, ADC measurement involves a sequence that can be readily appended to a standard abdominal MRI protocol; it requires no additional contrast medium and takes only a few minutes to acquire [16]. This implies that the method can be seamlessly integrated into existing clinical workflows. The ADC threshold value derived from this study offers clinicians a practical, objective, and quantitative reference point for ascites characterization.

Furthermore, the role of DWI may extend beyond that of a diagnostic tool. A potential application also exists in the monitoring of treatment response [5,8]. For instance, in a patient responding to intraperitoneal chemotherapy or systemic treatment, the cellularity and protein content within the ascites are expected to decrease concurrently with the reduction in peritoneal tumor burden [23]. This biological change would likely be reflected as an increase in ADC values. This may allow treatment efficacy to be determined non-invasively and before anatomical changes become apparent, demonstrating the potential of ADC as a prognostic marker.

The strengths of our study include a relatively large and well-defined patient cohort, the use of a robust reference standard such as cytology or histopathology for the diagnosis of malignancy, and the assessment of images by two experienced radiologists blinded to the final outcomes, supported by the demonstration of excellent inter-observer agreement. Even though the selection of the ‘darkest area’ within the ascites might appear subjective, the high reproducibility of the ADCmin measurements, confirmed by excellent ICC values (0.879), represents a significant strength of the present study. A standardized ROI size (50–100 mm^2^) was utilized to minimize partial volume averaging effects associated with very large ROIs, while maintaining sufficient pixel data to avoid the noise associated with very small ROIs. All patients were scanned on the same 1.5T MRI scanner (Siemens Aera) using a standard protocol, and all post-processing was performed on the same workstation, thereby eliminating inter-scanner and inter-vendor variability. Furthermore, the comprehensive quantitative analysis, incorporating both mean and minimum ADC values, and the direct comparison with traditional laboratory markers further augment the robustness of this study.

It has been stated in the literature that benign exudative ascites (e.g., tuberculosis, pancreatitis) may exhibit low ADC values that overlap with malignancy [21]. Although a formal subgroup analysis could not be performed due to the limited number of patients in these categories within our cohort, the ADC values were found to be 3229 ± 62.4 × 10^−6^ mm^2^/s for the pancreatitis subgroup (n = 10) and 3442 ± 20.5 × 10^−6^ mm^2^/s for the tuberculosis subgroup. Notably, these cases demonstrated ADC values higher than the established cut-off (≤2983 × 10^−6^ mm^2^/s), thereby allowing for their correct classification.

Nevertheless, our study is subject to several limitations. Its single-center and retrospective design may limit the generalizability of our findings. Since this is a single-center study, the results may reflect specific institutional characteristics and patient demographics, potentially limiting external validity. Multi-center validation studies are necessary to generalize these findings. The potential effects of different primary tumor types within the malignant ascites group on ADC values could not be examined due to an insufficient sample size for subgroup analyses. Because the benign group is heterogeneous in terms of etiology, different diseases can affect the ADC value. Future studies with larger subgroups are needed to analyze specific benign etiologies separately. DWI sequence is susceptible to bowel motion and magnetic susceptibility artifacts, which may have affected measurement accuracy in some patients. Although the number of such patients in our cohort was small, this represents a potential pitfall that could affect the method’s specificity, highlighting that clinical correlation remains essential.

Future studies should focus on validating our findings through multi-center investigations involving larger cohorts. The role of different b-values and more advanced diffusion models, such as Intravoxel Incoherent Motion (IVIM) and Diffusion Kurtosis Imaging (DKI), in the characterization of ascites warrants further exploration. Furthermore, studies examining the quantitative correlation between ADC values and specific biophysical parameters of the ascitic fluid will serve to further elucidate the fundamental principles of this method including cell count, protein concentration, and viscosity.

## 5. Conclusions

The present study indicates that quantitative ADC measurement derived from DWI is a valuable, non-invasive, reproducible, and reliable biomarker for the differentiation of benign and malignant ascites. ADC measurement possesses the potential to be integrated into existing diagnostic algorithms and to contribute significantly to the clinical decision-making process, especially in situations where invasive procedures are challenging or inconclusive.

## Figures and Tables

**Table 1:** Demographic and Clinical Characteristics of the Patient Groups.

	Group 1: Benign (n = 85)	Group 2: Malignant (n = 65)	*p*-Value
Age (year, mean ± SD)	66.2 ± 6.9	64.0 ± 6.0	0.094
Gender (Female, n [%])	37 (48.2%)	33 (56.9%)	0.378
**Primary Etiology (n [%])**			
**Group 1: Benign (n = 85)**		**Group 2: Malignant (n = 65)**	
Liver cirrhosis	46 (54.1%)	Ovarian cancer	27 (41.5%)
Congestive heart failure	16 (18.8%)	Gastric cancer	13 (20%)
Pancreatitis	10 (11.7%)	Colorectal cancer	13 (20%)
Nephrogenic ascites	10 (11.7%)	Pancreatic cancer	6 (9.2%)
Peritoneal Tuberculosis	2 (2.3%)	Cervical cancer	4 (6.1%)
Budd-Chiari Syndrome	1 (1.1%)	Primary peritoneal carcinomatosis	2 (3%)

**Table 2:** Between-Group Comparison of Laboratory Parameters.

Parameter (Mean ± SD)	Group 1: Benign (n = 85)	Group 2: Malignant (n = 65)	*p*-Value
Serum Albumin (g/dL)	3.01 ± 0.32	3.15 ± 0.28	0.14
Ascites Albumin (g/dL)	1.49 ± 0.35	2.71 ± 0.41	<0.001
SAAG (g/dL)	1.40 ± 0.30	0.72 ± 0.34	<0.001
Serum LDH (U/L)	207.07 ± 28.9	404 ± 77.7	<0.001
Ascites LDH (U/L)	112.2 ± 21.1	504.3 ± 77	<0.001
Serum Glucose (mg/dL)	110.5 ± 13.0	120.4 ± 9.6	0.08
Ascites Glucose (mg/dL)	107.1 ± 12.4	69.9 ± 5.9	<0.001

SAAG: Serum-Ascites Albumin Gradient; LDH: Lactate dehydrogenase; SD: Standard Deviation. *p*-values were obtained using Mann–Whitney U test.

**Table 3:** Comparison of ADC Values in the Benign and Malignant Ascites Groups (mean ± SD).

(×10^−6^ mm^2^/s)	Group 1: Benign (n = 85)	Group 2: Malignant (n = 65)	*p*-Value
ADCmean	3596 ± 239	3162 ± 204	0.006
ADCmin	3322 ± 218	2885 ± 148	0.0016

*p*-values were obtained using Mann–Whitney U test.

**Table 4:** Diagnostic Performance Analysis of ADC Parameters and SAAG.

Parameter	AUC (95% CI)	Optimal Cut-Off	Sensitivity (%) [95% CI]	Specificity (%) [95% CI]	PPV (%) [95% CI]	NPV (%) [95% CI]	Youden’s Index
ADCmean	0.877 (0.822–0.932)	3378 × 10^−6^ mm^2^/s	80 [68.2–88.9]	82.3 [72.5–89.7]	77.6 [68.3–84.7]	84.3 [76.6–89.8]	0.62
ADCmin	0.930 (0.892–0.968)	2983 × 10^−6^ mm^2^/s	81.5 [74.4–86.9]	85.8 [75.9–90.2]	81.5 [75.4–86.7]	85.8 [80.1–89.9]	0.67
SAAG	0.919 (0.874–0.965)	1.25 g/dL	72.2 [64.7–84.0]	92.3 [83.7–95.8]	95.5 [87.5–98.4]	74.7 [66.9–81.1]	0.64

ADCmean: Mean Apparent Diffusion Coefficient; ADCmin: Minimum Apparent Diffusion Coefficient; AUC: Area Under Curve; CI: Confidence Interval; PPV: Positive Predictive Value; NPV: Negative Predictive Value.

## Data Availability

The raw data supporting the conclusions of this article will be made available by the authors on request.
